# Self-perception of depressive symptoms, self-reported medical diagnosis, and associated factors: findings from the Brazilian Longitudinal Study of Aging, 2019-2021

**DOI:** 10.1590/S2237-96222026v35e20250158.en

**Published:** 2026-01-16

**Authors:** Ana Cristina Baggio, Gabriela Mahana, Cesar de Oliveira, Juliana Coelho de Campos, Daisson José Trevisol, Fabiana Schuelter-Trevisol, Eliane Traebert, Jefferson Traebert

**Affiliations:** 1 Universidade do Sul de Santa Catarina, Curso de Medicina, Palhoça, SC, Brasil; 2 University College London, Department of Epidemiology and Public Health, Londres, Reino Unido; 3 Universidade do Sul de Santa Catarina, Programa de Pós-Graduação em Ciências da Saúde, Palhoça, SC, Brasil

**Keywords:** Depressive Disorder, Aged, Self Report, Prevalence, Cross-Sectional Studies, Trastorno Depresivo, Anciano, Autoinforme, Prevalencia, Estudios Transversales

## Abstract

**Objectives:**

To estimate the prevalence of self-reported physician-diagnosed depression and to assess its association with self-perceived depressive symptoms, sociodemographic variables, physical activity, alcohol consumption, and smoking among Brazilian older adults.

**Methods:**

This study employed a cross-sectional design using data from the Brazilian Longitudinal Study of Aging. The outcome was the self-reported physician-diagnosed depression. Independent variables included sociodemographic factors, physical activity, alcohol consumption, and smoking. The association between the outcome and self-perception of depressive symptoms was evaluated.

**Results:**

Data from 6,872 older adults were included. The prevalence of self-reported physician-diagnosed depression was 12.2% (95% confidence interval - 95%CI 11.4; 13.0). Self-perceived depressive symptoms were reported by 15.6% (95%CI 14.7; 16.5). Self-reported physician-diagnosed depression was higher among females (prevalence ratio - PR 2.23; 95%CI 1.80; 2.89), individuals with up to eight years of schooling (PR 1.32; 95% CI 1.10; 1.61), and those who did not engage in physical activity (PR 1.32; 95% CI 1.10; 1.57).

**Conclusion:**

The prevalence of self-reported physician-diagnosed depression among Brazilian older adults was 12.2%, independently associated with female sex, lower schooling, and physical inactivity. However, the prevalence of self-perceived depressive symptoms was even higher and was also associated with self-reported physician-diagnosed depression. Self-report may be a valuable tool, reinforcing the need for a hybrid approach that combines it with objective methods when addressing depression among older people.

## Resumen

**Objetivos:**

Estimar la prevalencia del autorreporte de diagnóstico médico de depresión y evaluar su asociación con la autopercepción de síntomas depresivos, variables sociodemográficas, actividad física, consumo de bebidas alcohólicas y tabaquismo en ancianos brasileños.

**Métodos:**

Se realizó un estudio transversal con datos del Estudio Longitudinal de Salud de los Ancianos Brasileños. El autorreporte de diagnóstico médico de depresión fue el desenlace. Las variables independientes fueron sociodemográficas, actividad física, consumo de bebidas alcohólicas y tabaquismo. Se verificó la asociación entre el desenlace y el autorreporte de síntomas depresivos.

**Resultados:**

Se incluyeron datos de 6.872 ancianos. La prevalencia del autorreporte de diagnóstico médico de depresión fue del 12,2% (intervalo de confianza del 95% – IC95% 11,4; 13,0). La autopercepción de síntomas depresivos fue reportada por el 15,6% (IC95% 14,7; 16,5). El autorreporte de diagnóstico médico de depresión fue mayor en el sexo femenino (razón de prevalencia – RP 2,23; IC95% 1,80; 2,89), en personas con escolaridad de hasta ocho años de estudio (RP 1,32; IC95% 1,10; 1,61) y que no practicaban actividad física (RP 1,32; IC95% 1,10; 1,57).

**Conclusión:**

La prevalencia del autorreporte de diagnóstico médico de depresión fue del 12,2% en ancianos brasileños, asociada de forma independiente con el sexo femenino, menor escolaridad y no practicar actividad física. Sin embargo, la prevalencia de autopercepción de síntomas depresivos fue mayor, también asociada con el autorreporte de diagnóstico médico de depresión. El autorreporte puede ser valioso, lo que refuerza la necesidad de un enfoque híbrido que se combine con métodos objetivos para el abordaje de la depresión en ancianos.

**Ethical aspects**

This research respected ethical principles, having obtained the following approval data:

Research Ethics Committee: Centro de Pesquisas René Rachou

Opinion number: 886754

Approval date: 6/9/2017

Certificate of Submission for Ethical Appraisal: 34649814.3.0000.5091

Informed Consent Form: Obtained from all participants prior to data collection.

## Introduction 

The remarkable increase in life expectancy in recent decades has led to a significant global demographic transformation. This change is reflected in the age distribution, with a substantial increase in the proportion of people aged 60 years and older, surpassing other age groups. As of 2022, Brazil had approximately 30.6 million individuals aged 60 years or older, accounting for 15.1% of the total population (1).

Depression is a frequent condition among older adults (2), as advancing age is associated with an increase in depressive symptoms, often expressed through complaints related to various comorbidities (3). A large proportion of older adults report negative self-perception of health, which also affects their use of health services (4). Mood disorders are often underdiagnosed and undertreated, leading to increased functional dependence, social isolation, suicide risk, worse quality of life, and higher mortality (5).

In 2017, it was estimated that 50.0% of older adults with depression were not diagnosed by primary care professionals, as symptoms may resemble the natural process of aging (6). Among these symptoms, fatigue, sleep disturbances, lack of libido, memory problems, and irritability are the most frequently reported (7). Additionally, psychosocial perspectives are criteria that individualize human beings, and for this reason, the subjective analysis of feelings becomes valid and extremely important (8). For example, in some individuals, certain situations may trigger feelings of excessive worry or the “empty nest” syndrome. Hormonal changes related to menopause, as well as external factors such as retirement, may also contribute to the complexity of these cases (8).

Self-reported depressive symptoms, a common approach in mental health epidemiological surveys, have proven beneficial to estimate the prevalence of mental disorders in the population, especially in large-scale studies (9). Nevertheless, this strategy has important limitations, including the potential for bias related to unequal access to health services, the understanding of symptoms, and the prior recognition of a diagnosis by a health professional. These factors may influence both underreporting and overestimation of cases and must be considered when interpreting results (10).

On the other hand, studies based on the Brazilian National Health Survey (*Pesquisa Nacional de Saúde* - PNS) have demonstrated the relevance of combining self-reported physician diagnosis with depressive symptom screening through instruments such as the Patient Health Questionnaire-9 (PHQ-9). In the 2013 PNS, the prevalence of depressive symptoms (PHQ-9≥10) was found to be 9.7%, and that of major depression was 3.9% in the adult population. Among individuals with high PHQ-9 scores, only 7.2% reported having previously received a medical diagnosis, suggesting possible underdiagnosis (11). In the 2019 PNS, favorable screening rates of 10.8% (PHQ-9≥10) and a prevalence of 5.7% of major depression were observed, reinforcing the existence of clinically relevant symptoms in a significant portion of the population that may not have been formally diagnosed (12).

Despite increasing attention to the mental health of the older population, important gaps remain in the national literature regarding an integrated understanding of self-reported physician-diagnosed depression and self-perceived depressive symptoms among Brazilian older adults. Few studies have specifically explored depressive symptoms in this population, particularly in relation to behavioral and sociodemographic factors. Additionally, several studies have examined the potential discrepancies between self-reported physician diagnoses and the presence of subjective symptoms, which may reveal inequalities in access to diagnosis and treatment (13). In this context, this study aims to contribute by investigating, based on nationally representative data, the prevalence of self-reported depression and its association with multiple factors in Brazilian older adults, as well as by expanding the understanding of social and individual determinants of mental health in this population.

This study aimed to estimate the prevalence of self-reported physician-diagnosed depression and to assess its association with self-perceived depressive symptoms, sociodemographic variables, physical activity, alcohol consumption, and smoking among older persons.

## Methods 

### Study Design

This was a cross-sectional study.

### Setting 

This study used data from the Brazilian Longitudinal Study of Aging (ELSI-Brazil) (14). The survey, conducted between 2019 and 2021 by the Oswaldo Cruz Foundation/Minas Gerais and the Federal University of Minas Gerais, aimed to investigate the dynamics of aging in the Brazilian population and its determinants, as well as the demands of this population on social and health systems.

### Participants 

The study included data on older adults of both sexes who participated in ELSI-Brazil between 2019 and 2021, based on the publicly available database at https://elsi.cpqrr.fiocruz.br/. Permission to use these data was obtained through registration on the official study website.

### Variables 

The outcome was self-reported physician-diagnosed depression, investigated through the following question: “*Has a doctor ever told you that you have depression*?” Participants were also asked about the use of medications for treating depression. Self-reported depressive symptoms were assessed through questions related to feeling depressed, perceiving life as difficult, non-restorative sleep, happiness, loneliness, enjoyment of life, and feelings of sadness. 

Exposure variables included: sex, age, education level, marital status, being retired or receiving a pension, engagement in physical activity, alcohol consumption, and smoking.

### Data sources

All variables were obtained from the ELSI-Brazil (2019-2021) database. The interviews that generated the database were conducted at participants’ homes by trained researchers from the main study team, using a structured questionnaire with closed-ended questions. 

### Bias 

The ELSI-Brazil (2019-2021) study employed an inverse sampling design to mitigate nonresponse bias, eliminating the need to increase the sample size.

### Study size

This study included data from all older adults present in the ELSI-Brazil (2019-2021) database. The sample was designed to be representative of the Brazilian community-dwelling population aged 50 years or older. To ensure the representativeness of urban and rural areas in small, medium, and large municipalities, the sampling design employed a multistage model that combined stratification by municipality, census tract, and household. 

Municipalities were allocated into four strata according to population size: first stratum (≤26,700 inhabitants, encompassing 4,420 municipalities); second stratum (26,701-135,000 inhabitants, with 951 municipalities); third stratum (135,001-750,000 inhabitants, with 171 municipalities); and fourth stratum (>750,000 inhabitants, with 23 municipalities). All household residents aged 50 years or older in the selected households were eligible for the interview. 

The final sample consisted of participants from 70 municipalities distributed across the five regions of Brazil. Sampling weights were calculated to account for the differential probability of selection and differential nonresponse. Additional details on methodological aspects, including the sampling process, can be found in another publication (14).

### Statistical methods

The chi-square test was used to assess the association between self-reported physician-diagnosed depression and the following exposure variables: age (categorized according to the median of the distribution), sex, education level (illiterate, up to eight years of schooling, and more than eight years of schooling), marital status (with partner, without partner), physical activity (yes, no), smoking (yes, no), alcohol consumption (yes, no), and retirement/pension status (yes, no). 

To control for confounding, all variables with p<0.20 in the bivariate analysis were included in a multivariate model using Poisson regression with robust variance. Prevalence ratios (PR) and their respective 95% confidence intervals (95%CI) were estimated. 

The chi-square test was also used to assess the association between self-reported physician-diagnosed depression and self-reported depressive symptoms, assessed by questions related to feeling depressed, perceiving life as difficult, non-restorative sleep, happiness, loneliness, enjoyment of life, and feelings of sadness.

## Results 

A total of 6,872 individuals aged 60 years or older were included in the study. Of these, 59.5% were women. The mean age was 71.1 years (standard deviation 8.3); the median was 70.0 years; and the mode was 63.0 years. In the sample, 46.6% did not engage in physical activity, 18.0% consumed alcoholic beverages, and 10.8% were smokers (Table 1).

**Table 1:** Prevalence of depression based on self-reported physician diagnosis and its association with sociodemographic and behavioral variables among older adults in the Brazilian Longitudinal Study of Aging. Brazil, 2019-2021 (n=6,872)

Variables	Self-reported physician-diagnosed depression		
	Yes	No	p-value
	n (%)	n (%)	
**Sex** (n=6,872)			<0.001
Male	195 ( 7.0)	2,587 (93.0)	
Female	653 ( 16.0)	3,437 (84.0)	
**Median age** (years) (n=6,872)			0.001
60-70	500 ( 13.5)	3,197 (85.5)	
>70	348 ( 11.0)	2,827 (89.0)	
**Education level** (years) (n=6,783)			0.007
No schooling	133 (10.1)	1,180 (89.9)	
Up to 8	549 (13.3)	3,586 (86.7)	
>8	155 (11.6)	1,180 (88.4)	
**Marital status** (n=6,872)			0.001
Without partner	472 (13.7)	2,980 (86.3)	
With partner	376 (11.0)	3,044 (89.0)	
**Retirement/pension status** (n=6,859)			<0.001
No	271 (14.8)	1,564 (85.2)	
Yes	574 (11.4)	4,450 (88.6)	
**Physical activity** (n=6,767)			<0.001
No	444 (14.1)	2,711 (85.9)	
Yes	396 (11.0)	3,216 (89.0)	
**Consuming alcoholic beverages** (n=6,872)			<0.001
Yes	115 (9.3)	1,116 (90.7)	
No	730 (13.0)	4,891 (87.0)	
**Smoking habits** (n=6,872)			0.570
Yes	87 (11.7)	657 (88.3)	
No	761 (12.4)	5,367 (87.6)	

Results of the association analyses between self-reported physician-diagnosed depression and sociodemographic and behavioral variables were observed (Table 1). The prevalence of self-reported physician-diagnosed depression was 12.2% (n=842) (95%CI 11.4; 13.0). The report of antidepressant medication use was 8.1% (n=569) (95%CI 7.5; 8.7). Women, older adults aged 60–70 years, individuals with lower schooling, without a partner, and without retirement or pension benefits, more frequently reported physician-diagnosed depression. Prevalence was also higher among those who were physically inactive and those who did not consume alcoholic beverages.

Results of the association between self-reported depressive symptoms and physician-diagnosed depression were also identified (Table 2). Regarding self-perception of depressive symptoms reported by the study participants, 15.6% (95%CI 14.7; 16.5) stated that they felt depressed. A significant association was found between self-reported physician-diagnosed depression and self-perception of various depressive symptoms reported in the previous week. Older adults who reported feeling depressed (p-value<0.001), sad (p-value<0.001), lonely (p-value<0.001), lacking pleasure in life (p-value<0.001), experiencing non-restorative sleep (p-value<0.001), or who perceived difficulties in managing their daily tasks (p-value<0.001) were more frequently diagnosed with depression. Conversely, those who reported feeling happy or did not perceive negative changes in their emotional state showed a lower prevalence of depression diagnosis.

**Table 2:** Association between self-reported physician-diagnosed depression and self-perceived depressive symptoms reported most of the time in the past week among older adults in the Brazilian Longitudinal Study of Aging. Brazil, 2019-2021 (n=6,082)

Self-perceived depressive symptoms	Self-reported physician-diagnosed depression			
	Yes	No	Total	p-value
	n (%)	n (%)	n (%)	
**Felt depressed** (n=6,082)				<0.001
Yes	355 (37.3)	596 (62.7)	951 (15.6)	
No	374 (7.3)	4,757 (92.7)	5,131 (84.4)	
**Felt that things were more difficult than before** (n=6,065)				<0.001
Yes	390 (22.1)	1,374 (77.9)	1,764 (29.1)	
No	337 (7.8)	3,964 (92.2)	4,301 (70.9)	
**Experienced non-restorative sleep** (n=6,068)				<0.001
Yes	336 (22.1)	1,183 (77.9)	1,519 (25.0)	
No	391 (8.6)	4,158 (91.4)	4,549 (75.0)	
**Felt happy** (n=6,038)				<0.001
No	284 (25.9)	814 (74.1)	1,098 (18.2)	
Yes	439 (8.9)	4,501 (91.1)	4,940 (81.8)	
**Felt lonely** (n=6,048)				<0.001
Yes	315 (26.0)	896 (74.0)	1,211 (20.0)	
No	408 (8.4)	4,429 (91.6)	4,837 (80.0)	
**Enjoyed/felt pleasure in life** (n=6,007)				<0.001
No	269 (22.2)	940 (77.8)	1,209 (20.1)	
Yes	453 (9.4)	4,345 (90.6)	4,798 (79.9)	
**Felt sad** (n=6,044)				<0.001
Yes	343 (27.6)	901 (72.4)	1,244 (20.6)	
No	381 (7.9)	4,419 (92.1)	4,800 (79.4)	
**Felt unable to carry on with things** (n=6,023)				<0.001
Yes	348 (22.1)	1,224 (77.9)	1,572 (26.1)	
No	372 (8.4)	4,079 (91.6)	4,451 (73.9)	

The results of the multivariate analysis, which mutually adjusted for sociodemographic and behavioral variables, are presented for self-reported physician-diagnosed depression (Table 3). The self-reported physician-diagnosed depression was independently higher among females (PR 2.23; 95%CI 1.80; 2.89; p-value<0.001), individuals with up to eight years of schooling (PR 1.32; 95%CI 1.10; 1.61; p-value 0.004), and those who did not engage in physical activity (PR 1.32; 95%CI 1.10; 1.57; p-value 0.002).

**Table 3:** Unadjusted and adjusted prevalence ratios (PR) and 95% confidence intervals (95%CI) for self-reported physician-diagnosed depression in Brazilian older adults according to study variables. Brazil, 2019-2021 (n=6,872)

Variables	Self-reported physician-diagnosed depression			
	Unadjusted PR (95%IC)	p-value	Adjusted PR (95%IC)	p-value
Sex		<0.001		<0.001
Male	1.00		1.00	
Female	2.34 (1.90; 2.89)		2.23 (1.80; 2.89)	
**Median age** (years)		0.653		0.173
60-70	1.00		1.00	
>70	1.04 (0.88; 1.26)		1.12 (0.95; 1.34)	
**Education level** (years)				
No schooling	1.00		1.00	
Up to 8	1.35 (1.12; 1.62)	0.001	1.32 (1.10; 1.61)	0.004
>8	1.19 (0.89; 1.60)	0.240	1.13 (0.86; 1.51)	0.366
**Marital status**				
Without partner	1.35 (1.13; 1.61)	0.001	1.09 (0.92; 1.29)	0.335
With partner	1.00		1.00	
**Retirement/pension status**				
No	1.28 (1.06; 1.52)	0.012	1.11 (0.91; 1.35)	0.303
Yes	1.00		1.00	
**Physical activity**		0.010		0.002
No	1.27 (1.06; 1.52)		1.32 (1.10; 1.57)	
Yes	1.00		1.00	
**Alcoholic beverage consumption**		0.001		0.205
Yes	0.66 (0.52; 0.84)		0.86 (0.68; 1.08)	
No	1.00		1.00	

## Discussion 

The findings of this study suggest that a medical diagnosis of depression among older persons may not fully capture their everyday experience of symptoms, indicating potential shortcomings in clinical recognition or access to mental health care. The predominance of symptoms among specific population segments, such as women, individuals with intermediate schooling, and those who are physically inactive, points to the influence of social and behavioral inequalities on the expression and management of mental health among the aged. These results reinforce the need for more sensitive and integrated approaches to identifying and monitoring depressive conditions in this age group, considering the multiple dimensions that affect detection and treatment.

The results presented here must be interpreted critically. The absence of a validated instrument to assess the primary outcome can be considered a significant limitation. The fact that the guiding question for depression diagnosis is based on the patient’s self-report of medical information may also lead to information bias, as it is impossible to identify which diagnostic criteria were used. 

The investigation of self-reported depressive symptoms does not necessarily refer to a depressive disorder according to medical criteria or the use of validated scales. However, it is emphasized that the relationship between these variables is precisely the essence of this work. Another limitation of this study is the non-inclusion of clinical comorbidities and social support in this analysis, which are known to influence depression in older adults.

Beyond the perspective of self-reported physician-diagnosed depression, a distinctive feature of this study was its use of self-reported depressive symptoms by the older adults themselves. The analysis of an older individual’s self-perception can be a plausible indicator of the need for follow-up care. The benefits of this approach are independent of the use of geriatric scales or normative processes. 

Specific determinant variables were also used to elucidate, on an objective level, the prevalence of depression in relation to the sociodemographic profile of aged people, and on a subjective level, how these individuals experience depressive symptoms in their daily lives. This study demonstrates a higher prevalence of self-reported physician-diagnosed depression among older adults who are female, have up to eight years of schooling, and have a sedentary lifestyle. Correlations were established between feelings of loneliness, happiness, pleasure in life, and restorative sleep with the self-report of a medical depression diagnosis in this population. These findings are consistent with those described in other studies (15-16).

A higher prevalence of self-reported medical diagnosis of major depression was found in women. Being female is a well-known and well-documented risk factor for depression (2,17). Biological factors, such as hormonal fluctuations during the menstrual cycle, pregnancy, postpartum period, and menopause, are associated with an increased risk of depression. Psychosocially, women are more vulnerable to stressors related to social roles, victimization, and disadvantaged social status, which may further increase their susceptibility to depression (18). It can also be stated that women are more likely than men to recognize psychiatric problems, discuss these issues with others, and voluntarily initiate treatment (19).

One lifestyle habit investigated in this study was the practice of physical activity. A higher prevalence of self-reported physician-diagnosed depression was observed among those who did not engage in physical activity. Other authors reinforce that a sedentary lifestyle can lead to a decline in activities of daily living, which in turn interferes with quality of life. At the same time, regular exercise is a protective factor against depression in the aged (20,21).

Lower levels of schooling are associated with increased depressive symptoms. This is partly because individuals with less education tend to have less access to health services and participate less in activities that stimulate cognitive functions and social interaction. Furthermore, education has been recognized as a directly protective factor for mental health (22). 

In this study, it was observed that older persons with up to eight years of schooling had a higher prevalence of depression compared to those with no formal education. This finding, although seemingly contradictory to the literature, may reflect a mismatch between expectations and opportunities experienced by individuals with intermediate schooling. Despite having some access to formal education, they may not have seen significant improvements in their socioeconomic conditions. This situation can accentuate feelings of frustration and emotional vulnerability. These results underscore the importance of conducting contextual and in-depth analyses when examining the relationship between education and mental health in the elderly population.

The discussion on depression among older adults is complex. The use of self-reports to assess health conditions in the elderly population offers benefits but also presents significant challenges. The self-report allows older people to directly express their perceptions of health and well-being, which is valuable since many symptoms may be underdiagnosed through traditional clinical assessments (23). Objectively, depression in the elderly population can be quantified using validated scales, such as the Geriatric Depression Scale, which determines specific criteria for suspected diagnosis of depression or even the need for referral to a psychiatrist (24). 

Intriguingly, this study demonstrated high rates of depressive symptoms among older people who had no self-reported physician diagnosis of depression. Among the 15.9% of older adults who felt depressed, only 37.3% reported a physician’s diagnosis of depression. It implies that 62.7% felt depressed but did not report a medical diagnosis. 

High proportions were also identified for other questions, such as feeling that things were more difficult than they used to be, where 77.9% of a total of 29.1% also did not report a medical diagnosis. This pattern persisted for the other questions related to the investigated feelings. These questions do not necessarily directly reflect depression, but can be considered potential indicators for Primary Care services to intervene early to prevent the occurrence of depression. Categorizing such symptoms into scores and applying them in daily practice can be challenging (25). 

To improve the management of depression among the aged, healthcare professionals must combine self-report with objective assessments using validated scales and more integrated diagnostic methods, which may also include imaging exams and biochemical tests. A hybrid approach could lead to better identification and treatment of disorders often neglected in this age group (23).

The difficulty in reporting a physician-diagnosed depression may be related to the aged adopting a self-help approach, hypothetically preferring to solve their problems alone, aligning with cultural narratives that value self-sufficiency and resilience. This behavior is reinforced by the view that mental health problems are signs of personal weakness, which hinders the acceptance of diagnoses and treatments (26). The potential lack of accessibility to public health resources among older adults may also interfere and should be explicitly addressed in public policy planning (15).

When clarifying the importance of diagnosis and intervention measures, it is fundamental that healthcare professionals understand the need to avoid undertreating the elderly population. This study revealed significant numbers of patients already diagnosed with depression who continued to present depressive symptoms. This situation may occur due to the overlap of depressive symptoms with other health conditions (27). 

Self-rated health is one of the indicators used in research involving older adults, and its relevance stems from the fact that negative self-rated health can be a strong and consistent predictor of mortality (28). In 2011, in Pelotas, Rio Grande do Sul, 46.9% of the elderly population rated their health as fair or poor, and 66.6% of the older persons with depressive symptoms reported negative perceptions about their health (29).

Healthcare professionals and support networks must be aware of conditions that increase the risk of developing depression, as well as its signs and symptoms, dispelling the perception that these aspects are natural parts of the aging process. These professionals should consider the set of factors that impact the lives and mental health of older adults and adopt more comprehensive approaches that recognize the multicausality of diseases (30), including depression. Therefore, it becomes crucial to incorporate social and environmental issues into the analysis to gain a more comprehensive understanding of the reality faced by this population, in addition to directing efforts towards promoting public policies and deepening studies focused on the fastest-growing age group in the population pyramid.

In conclusion, there is a relevant discrepancy between self-reported physician-diagnosed depression and the self-perception of depressive symptoms among Brazilian older adults. The proportion of people who report feeling depressed exceeds that of those who report having received a medical diagnosis, highlighting a potential gap in the clinical recognition of the condition. Factors such as female sex, sedentary lifestyle, and lower schooling were associated with a higher prevalence of depressive symptoms. Self-report, although valuable, may be limited by underreporting or the self-concept of the aged, reinforcing the need for a hybrid approach that combines self-report and objective methods for adequate diagnosis and management of depression.
