# Supplementary material for: Self-perception of depressive symptoms, self-reported medical diagnosis, and associated factors: findings from the Brazilian Longitudinal Study of Aging, 2019-2021
Source: Epidemiol Serv Saude. 2026 Jan 16;35:e20250158. doi: 10.1590/S2237-96222026v35e20250158.en (PMC12810687; doi:10.1590/S2237-96222026v35e20250158.en)
Supplement: Translation

# Translation: Autopercepção de sintomas depressivos, autorrelato de diagnóstico médico e fatores associados: achados do Estudo Longitudinal da Saúde dos Idosos Brasileiros, 2019-2021

**DOI:** 10.1590/S2237-96222026v35e20250158.pt

**Authors:** Ana Cristina Baggio, Gabriela Mahana, Cesar de Oliveira, Juliana Coelho de Campos, Daisson José Trevisol, Fabiana Schuelter-Trevisol, Eliane Traebert, Jefferson Traebert

**Affiliations:** 1 Universidade do Sul de Santa Catarina, Curso de Medicina, Palhoça, SC, Brasil; 2 University College London, Department of Epidemiology and Public Health, Londres, Reino Unido; 3 Universidade do Sul de Santa Catarina, Programa de Pós-Graduação em Ciências da Saúde, Palhoça, SC, Brasil

**Keywords:** Transtornos Depressivos, Idosos, Autorrelato, Prevalência, Estudos Transversais

## Abstract

**Objetivos:**

Estimar a prevalência de autorrelato de diagnóstico médico de depressão e avaliar sua associação com autopercepção de sintomas depressivos, variáveis sociodemográficas, atividade física, consumo de bebida alcoólica e tabagismo em idosos brasileiros.

**Métodos:**

Tratou-se de estudo transversal com dados do Estudo Longitudinal de Saúde dos Idosos Brasileiros. O autorrelato de diagnóstico médico de depressão foi o desfecho. As variáveis independentes foram sociodemográficas, atividade física, consumo de bebida alcoólica e tabagismo. Verificou-se a associação entre o desfecho e o autorrelato de sintomas depressivos.

**Resultados:**

Foram incluídos dados de 6.872 idosos. A prevalência de autorrelato de diagnóstico médico de depressão foi 12,2% (intervalo de confiança de 95% – IC95% 11,4; 13,0). Autopercepção de sintomas depressivos foi relatado por 15,6% (IC95% 14,7; 16,5). O autorrelato de diagnóstico médico de depressão foi maior no sexo feminino (razão de prevalência – RP 2,23; IC95% 1,80; 2,89), em pessoas com escolaridade até oito anos de estudo (RP 1,32; IC95% 1,10; 1,61) e que não praticavam atividade física (RP 1,32; IC95% 1,10; 1,57).

**Conclusão:**

A prevalência de autorrelato de diagnóstico médico de depressão foi 12,2% em idosos brasileiros, associada de forma independente com sexo feminino, menor escolaridade e não praticar a atividade física. Entretanto, a prevalência de autopercepção de sintomas depressivos foi maior, também associada com o autorrelato de diagnóstico médico de depressão. O autorrelato pode ser valioso, o que reforça a necessidade de abordagem híbrida que combine com métodos objetivos para a abordagem da depressão em idosos.

**Aspectos éticos**

A presente pesquisa respeitou os princípios éticos, obtendo os seguintes dados de aprovação:

Comitê de ética em pesquisa: Centro de Pesquisas René Rachou

Número do parecer: 886.754

Data de aprovação: 6/9/2017

Certificado de apresentação de apreciação ética: 34649814.3.0000.5091

Registro do consentimento livre e esclarecido: Obtido de todos os participantes antes da coleta.

## Introdução 

O aumento notável na expectativa de vida nas últimas décadas provocou grande transformação demográfica global. Isso se reflete na distribuição etária, com crescimento expressivo da proporção de pessoas com 60 anos ou mais, superando outras faixas etárias. No Brasil, em 2022, havia cerca de 30,6 milhões de indivíduos com 60 anos ou mais, o que representava 15,1% da população total (1).

A depressão destaca-se como agravo frequente entre idosos (2), já que, com o avançar da idade, há aumento na manifestação de sintomas depressivos, muitas vezes expressos por meio de queixas relacionadas a diversas comorbidades (3). Grande parte dos idosos tem a autopercepção negativa da saúde, fato que também impacta a busca de serviços de saúde (4). Os transtornos de humor são frequentemente subdiagnosticados e subtratados, o que causa aumento da dependência funcional, isolamento social, risco de suicídio, piora da qualidade de vida e aumento da mortalidade (5).

Em 2017, estimou-se que 50,0% dos idosos com depressão não foram diagnosticados pelos profissionais de saúde na Atenção Primária, pois os sintomas podem ser assemelhar ao processo natural do envelhecimento (6). Entre tais sintomas, queixas de fadiga, sono, falta de libido, problemas de memória e irritabilidade são os mais frequentemente relatados (7). Além disso, as perspectivas psicossociais são critérios que individualizam os seres humanos e, por isso, a análise subjetiva dos sentimentos torna-se válida e extremamente importante (8). Por exemplo, em algumas pessoas, determinadas situações podem despertar sentimentos de preocupação excessiva ou a sensação de ninho vazio. Alterações hormonais relacionadas à menopausa, bem como fatores externos, como a aposentadoria, também podem contribuir para a complexidade desses casos (8).

O autorrelato de sintomas depressivos, abordagem comum em inquéritos epidemiológicos em saúde mental, tem se mostrado útil para estimar a prevalência de transtornos mentais na população, especialmente em estudos de grande escala (9). No entanto, essa estratégia apresenta limitações importantes, como a possibilidade de viés relacionado ao acesso desigual aos serviços de saúde, à compreensão dos sintomas e ao reconhecimento prévio de diagnóstico por um profissional de saúde. Tais fatores podem influenciar tanto a subnotificação quanto a superestimação dos casos e devem ser considerados na interpretação dos resultados (10).

Por outro lado, estudos baseados na Pesquisa Nacional de Saúde (PNS) têm demonstrado a relevância de combinar o autorrelato de diagnóstico médico com a triagem de sintomas depressivos por meio de escalas como a Patient Health Questionnaire-9 (PHQ-9). A partir da PNS 2013, verificou-se a prevalência de 9,7% de sintomas depressivos (PHQ-9≥10) e de 3,9% de depressão maior na população adulta. Entre os indivíduos com pontuação elevada na PHQ-9, apenas 7,2% relataram ter recebido diagnóstico médico prévio, o que indicou possível subdiagnóstico (11). Já na PNS 2019, observaram-se taxa de triagem positiva de 10,8% (PHQ-9≥10) e prevalência de 5,7% de depressão maior, o que reforçou a existência de sintomas clínicos relevantes em parcela significativa da população, que pôde não ter sido diagnosticada normativamente (12).

Apesar da crescente atenção à saúde mental da população idosa, ainda há lacunas importantes na literatura nacional quanto à compreensão integrada do autorrelato de diagnóstico médico de depressão e da autopercepção de sintomas depressivos em idosos brasileiros. Poucos trabalhos exploraram de forma específica os sintomas depressivos na população idosa, especialmente em associação a fatores comportamentais e sociodemográficos. Além disso, é limitado o número de estudos que discutem as possíveis discrepâncias entre o diagnóstico médico autorreferido e a presença de sintomas subjetivos, o que pode revelar desigualdades no acesso a diagnóstico e tratamento (13). Nesse sentido, este estudo pretende contribuir ao investigar, com base em dados nacionais representativos, a prevalência de depressão autorreferida e sua associação com múltiplos fatores em idosos brasileiros, além de ampliar o entendimento sobre determinantes sociais e individuais da saúde mental nessa população.

Este trabalho tem como objetivos estimar a prevalência de autorrelato de diagnóstico médico de depressão e avaliar sua associação com autopercepção de sintomas depressivos, variáveis sociodemográficas, atividade física, consumo de bebida alcoólica e tabagismo em idosos.

## Métodos 

### Delineamento do estudo

Tratou-se de estudo de delineamento transversal.

### Contexto 

Este trabalho utilizou dados do Estudo Longitudinal de Saúde dos Idosos Brasileiros (ELSI-Brasil) (14). Este estudo foi realizado no período 2019-2021 pela Fundação Oswaldo Cruz/Minas Gerais e pela Universidade Federal de Minas Gerais e teve como objetivos investigar a dinâmica do envelhecimento da população brasileira e seus determinantes, assim como a demanda dessa população para os sistemas sociais e de saúde.

### Participantes 

Este estudo abrangeu dados de idosos de ambos os sexos que participaram do ELSI-Brasil durante o período 2019-2021, a partir do banco de dados de domínio público, disponível em: https://elsi.cpqrr.fiocruz.br/. A permissão para utilizar esses dados foi obtida por meio de cadastro na página oficial do estudo.

### Variáveis 

O desfecho foi o autorrelato de diagnóstico médico de depressão investigado com a seguinte pergunta: “Algum médico já disse que o senhor tem depressão?”. Perguntou-se também sobre o uso de medicamentos para tratamento da depressão. O autorrelato de sintomas depressivos foi aferido por questões relacionadas a sentir-se deprimido, sentimento de dificuldade da vida, sono não reparador, felicidade, solidão, prazer pela vida e sentimento de tristeza. 

As variáveis de exposição foram: sexo, idade, escolaridade, situação conjugal, ser aposentado(a) ou receber pensão, praticar atividade física, consumo de bebida alcoólica e tabagismo.

### Fontes de dados

Todas as variáveis foram obtidas por meio do banco de dados do estudo ELSI-Brasil (2019-2021). As entrevistas que geraram o banco foram realizadas pela equipe de pesquisadores do estudo principal, previamente capacitados para essa tarefa, de forma domiciliar, por meio de aplicação de um questionário individual com perguntas fechadas. 

### Viés 

O estudo ELSI-Brasil (2019-2021) utilizou um desenho amostral com seleção inversa para mitigar o viés de não resposta, sem a necessidade de ampliar o tamanho da amostra.

### Tamanho do estudo

Este estudo utilizou dados de todos os idosos presentes no banco de dados do ELSI-Brasil (2019-2021). A amostra desse estudo foi desenhada para ser representativa da população brasileira residente na comunidade com 50 anos ou mais. Para garantir a representatividade das áreas urbanas e rurais de municípios de pequeno, médio e grande portes, o desenho amostral adotou um modelo por estágios, combinando estratificação dos municípios, setores censitários e domicílios. 

Os municípios foram alocados em quatro estratos, de acordo com o tamanho da população: primeiro estrato (≤26.700 habitantes, abrangendo 4.420 municípios); segundo estrato (26.701-135.000 habitantes, com 951 municípios); terceiro estrato (135.001-750.000 habitantes, com 171 municípios); e quarto estrato (>750 mil habitantes, com 23 municípios). Todos os residentes nos domicílios selecionados com 50 anos ou mais foram elegíveis para a entrevista. 

A amostra final foi composta por participantes de 70 municípios distribuídos nas cinco regiões do Brasil. Pesos amostrais foram calculados para considerar a probabilidade diferencial de seleção e a não resposta diferencial. Detalhes adicionais sobre outros aspectos metodológicos, incluindo o processo de amostragem, podem ser encontrados em outra publicação (14).

### Métodos estatísticos

O teste do qui-quadrado foi utilizado para testar associação entre o relato de diagnóstico médico de depressão e as seguintes variáveis de exposição: idade (categorizada na mediana da distribuição), sexo, nível de escolaridade (analfabeto, até oito anos completos e mais de oito anos completos de estudo), situação conjugal (com companheiro, sem companheiro), prática de atividade física (sim, não), tabagismo (sim, não), consumo de bebida alcoólica (sim, não) e receber aposentadoria/pensão (sim, não). 

Para controle de confundimento, todas as variáveis com p-valor<0,20 na análise bivariada foram incluídas em um modelo multivariado, utilizando regressão de Poisson com estimador robusto. Foram estimadas razões de prevalência (RP) e respectivos intervalos de confiança de 95% (IC95%). 

O teste do qui-quadrado foi utilizado também para testar associação entre o relato de diagnóstico médico de depressão e o autorrelato de sintomas depressivos, aferido por questões relacionadas a sentir-se deprimido, sentimento de dificuldade da vida, sono não reparador, felicidade, solidão, prazer pela vida e sentimento de tristeza.

## Resultados 

Foram incluídas no estudo 6.872 pessoas acima de 60 anos de idade. Desse total, 59,5% eram mulheres. A média de idade foi 71,1 anos (desvio-padrão 8,3); mediana, 70,0 anos; e moda, 63,0 anos. Da amostra, 46,6% não praticavam atividades físicas, 18,0% consumiam bebidas alcoólicas e 10,8% eram tabagistas (Tabela 1).

**Tabela 1:** Prevalência de depressão segundo autorrelato de diagnóstico médico e sua associação com variáveis sociodemográficas e comportamentais em idosos brasileiros do Estudo Longitudinal da Saúde dos Idosos Brasileiros. Brasil, 2019-2021 (n=6.872)

Variáveis	Autorrelato de diagnóstico médico de depressão		
	Sim	Não	p-valor
	n (%)	n (%)	
**Sexo** (n=6.872)			<0,001
Masculino	195 (7,0)	2.587 (93,0)	
Feminino	653 (16,0)	3.437 (84,0)	
**Idade mediana** (anos) (n=6.872)			0,001
60-70	500 (13,5)	3.197 (85,5)	
>70	348 (11,0)	2.827 (89,0)	
**Escolaridade** (anos) (n=6.783)			0,007
Não escolarizado	133 (10,1)	1.180 (89,9)	
Até 8	549 (13,3)	3.586 (86,7)	
>8	155 (11,6)	1.180 (88,4)	
**Situação conjugal** (n=6.872)			0,001
Sem companheiro/a	472 (13,7)	2.980 (86,3)	
Com companheiro/a	376 (11,0)	3.044 (89,0)	
**Aposentadoria/pensão** (n=6.859)			<0,001
Não	271 (14,8)	1.564 (85,2)	
Sim	574 (11,4)	4.450 (88,6)	
**Atividade física** (n=6.767)			<0,001
Não	444 (14,1)	2.711 (85,9)	
Sim	396 (11,0)	3.216 (89,0)	
**Consumo de bebida alcoólica** (n=6.872)			<0,001
Sim	115 (9,3)	1.116 (90,7)	
Não	730 (13,0)	4.891 (87,0)	
**Tabagismo** (n=6.872)			0,570
Sim	87 (11,7)	657 (88,3)	
Não	761 (12,4)	5.367 (87,6)	

Observaram-se os resultados dos estudos de associação entre o autorrelato de diagnóstico médico de depressão e as variáveis sociodemográficas e comportamentais (Tabela 1). A prevalência de autorrelato de diagnóstico médico de depressão foi 12,2% (n=842) (IC95% 11,4; 13,0). O relato de uso de medicação para depressão foi 8,1% (n=569) (IC95% 7,5; 8,7). Mulheres, idosos com idade entre 60 e 70 anos, pessoas com menor escolaridade, sem companheiro(a) e sem aposentadoria ou pensão relataram diagnóstico médico de depressão com mais frequência. A prevalência também foi maior entre aqueles fisicamente inativos e que não consomem bebidas alcoólicas.

Foram apontados os resultados do estudo de associação entre os sintomas depressivos relatados pelos entrevistados e o relato de diagnóstico médico de depressão (Tabela 2). No que diz respeito à autopercepção de sintomas depressivos relatados pelos indivíduos estudados, 15,6% (IC95% 14,7; 16,5) afirmaram sentir-se deprimidos. Houve associação significativa entre o autorrelato de diagnóstico médico de depressão e a autopercepção de diversos sintomas depressivos relatados na última semana. Idosos que se sentiram deprimidos (p-valor<0,001), tristes (p-valor<0,001), solitários (p-valor<0,001), sem prazer pela vida (p-valor<0,001), com sono não reparador (p-valor<0,001) ou que perceberam dificuldade em lidar com as próprias tarefas (p-valor<0,001) relataram mais frequentemente diagnóstico de depressão. De forma oposta, aqueles que se sentiram felizes ou não perceberam mudanças negativas em seu estado emocional apresentaram menor prevalência de diagnóstico.

**Tabela 2:** Associação entre autorrelato de diagnóstico médico de depressão e autopercepção de sintomas depressivos relatados na última semana na maior parte do tempo em idosos brasileiros do Estudo Longitudinal da Saúde dos Idosos Brasileiros. Brasil, 2019-2021 (n=6.082)

Autopercepção de sintomas depressivos	Autorrelato de diagnóstico médico de depressão			
	Sim	Não	Total	p-valor
	n (%)	n (%)	n (%)	
**Sentiu-se deprimido** (n=6.082)				<0,001
Sim	355 (37,3)	596 (62,7)	951 (15,6)	
Não	374 (7,3)	4.757 (92,7)	5.131 (84,4)	
**Sentiu que as coisas estavam mais difíceis do que antes** (n=6.065)				<0,001
Sim	390 (22,1)	1.374 (77,9)	1.764 (29,1)	
Não	337 (7,8)	3.964 (92,2)	4.301 (70,9)	
**Sentiu sono não reparador** (n=6.068)				<0,001
Sim	336 (22,1)	1.183 (77,9)	1.519 (25,0)	
Não	391 (8,6)	4.158 (91,4)	4.549 (75,0)	
**Sentiu-se feliz** (n=6.038)				<0,001
Não	284 (25,9)	814 (74,1)	1.098 (18,2)	
Sim	439 (8,9)	4.501 (91,1)	4.940 (81,8)	
**Sentiu-se solitário** (n=6.048)				<0,001
Sim	315 (26,0)	896 (74,0)	1.211 (20,0)	
Não	408 (8,4)	4.429 (91,6)	4.837 (80.0)	
**Aproveitou/sentiu prazer pela vida** (n=6.007)				<0,001
Não	269 (22,2)	940 (77,8)	1.209 (20,1)	
Sim	453 (9,4)	4.345 (90,6)	4.798 (79,9)	
**Sentiu-se triste** (n=6.044)				<0,001
Sim	343 (27,6)	901 (72,4)	1.244 (20,6)	
Não	381 (7,9)	4.419 (92,1)	4.800 (79,4)	
**Sentiu que não conseguiria levar adiante as suas coisas** (n=6.023)				<0,001
Sim	348 (22,1)	1.224 (77,9)	1.572 (26,1)	
Não	372 (8,4)	4.079 (91,6)	4.451 (73,9)	

Observaram-se os resultados da análise multivariada para autorrelato de diagnóstico médico de depressão e variáveis sociodemográficas e comportamentais, controladas entre si (Tabela 3). O relato de diagnóstico médico de depressão foi maior no sexo feminino (RP 2,23; IC95% 1,80; 2,89; p-valor<0,001), em pessoas com escolaridade até oito anos (RP 1,32; IC95% 1,10; 1,61; p-valor 0,004) e que não praticavam atividade física (RP 1,32; IC95% 1,10; 1,57; p-valor 0,002) de forma independente.

**Tabela 3:** Razões de prevalência (RP) brutas e ajustadas e intervalos de confiança de 95% (IC95%) do autorrelato de diagnóstico médico de depressão em idosos brasileiros pelas variáveis do estudo. Brasil, 2019-2021 (n=6.872)

Variáveis	Autorrelato de diagnóstico médico de depressão			
	RP bruta (IC95%)	p-valor	RP ajustada (IC95%)	p-valor
Sexo		<0,001		<0,001
Masculino	1,00		1,00	
Feminino	2,34 (1,90; 2,89)		2,23 (1,80; 2,89)	
**Idade mediana** (anos)		0,653		0,173
60-70	1,00		1,00	
>70	1,04 (0,88; 1,26)		1,12 (0,95; 1,34)	
**Escolaridade** (anos)				
Não escolarizado	1,00		1,00	
Até 8	1,35 (1,12; 1,62)	0,001	1,32 (1,10; 1,61)	0,004
>8	1,19 (0,89; 1,60)	0,240	1,13 (0,86; 1,51)	0,366
**Situação conjugal**				0,335
Sem companheiro/a	1,35 (1,13; 1,61)	0,001	1,09 (0,92; 1,29)	
Com companheiro/a	1,00		1,00	
Aposentadoria/pensão				0,303
Não	1,28 (1,06; 1,52)	0,012	1,11 (0,91; 1,35)	
Sim	1,00		1,00	
**Atividade física**		0,010		0,002
Não	1,27 (1,06; 1,52)		1,32 (1,10; 1,57)	
Sim	1,00		1,00	
**Consumo bebida alcoólica**		0,001		0,205
Sim	0,66 (0,52; 0,84)		0,86 (0,68; 1,08)	
Não	1,00		1,00	

## Discussão 

Os achados deste estudo sugerem que o diagnóstico médico de depressão em idosos pode não captar integralmente a vivência cotidiana dos sintomas, o que indica possíveis falhas no reconhecimento clínico ou no acesso ao cuidado em saúde mental. A predominância de sintomas em determinados segmentos da população, como mulheres, pessoas com escolaridade intermediária e aquelas fisicamente inativas, aponta para a influência de desigualdades sociais e comportamentais na expressão e no manejo da saúde mental em idosos. Esses resultados reforçam a necessidade de abordagens mais sensíveis e integradas para a identificação e o acompanhamento de quadros depressivos nessa faixa etária, considerando as múltiplas dimensões que afetam a detecção e o tratamento.

Os resultados aqui apresentados devem ser observados de forma crítica. A ausência de um instrumento validado para aferir o desfecho principal pode ser considerada uma importante limitação. O fato de a pergunta norteadora do diagnóstico de depressão ser o relato do paciente em relação à informação médica pode trazer também viés de informação, uma vez que não é possível identificar quais critérios foram utilizados para o diagnóstico. 

A investigação do autorrelato de sintomas depressivos não necessariamente se refere ao transtorno depressivo, de acordo com o critério médico ou o uso de escalas validadas. Todavia, ressalta-se que justamente a relação entre essas variáveis é que constituem a essência deste trabalho. Outra limitação deste estudo reside à não inclusão de comorbidades clínicas e suporte social nesta análise, conhecidos por influenciar a depressão em idosos.

Além da perspectiva do autorrelato de diagnóstico médico de depressão, um diferencial deste estudo se deu pela utilização do autorrelato dos sintomas depressivos pelos idosos. A análise da percepção individual do idoso sobre si mesmo pode ser um indicativo plausível de necessidade de acompanhamento. Os benefícios dessa abordagem independem do uso de escalas geriátricas ou processos normativos. 

Também foram utilizadas variáveis determinantes específicas para elucidar, em nível objetivo, a prevalência da depressão em relação ao perfil sociodemográfico dos idosos e, em nível subjetivo, a forma como esses indivíduos se relacionam cotidianamente com sintomas depressivos. Este estudo demonstra maior prevalência de autorrelato de diagnóstico médico de depressão em idosos do sexo feminino, com escolaridade até oito anos e estilo de vida sedentário. Estabelecem-se correlações entre sentimentos de solidão, felicidade, prazer pela vida e sono reparador com o relato de diagnóstico médico de depressão nessa população. Estes achados estão em consonância com o que é descrito em outras pesquisas (15-16).

Foi encontrada maior prevalência de relato de diagnóstico médico de depressão maior em mulheres. Ser do sexo feminino é um fator de risco para depressão bastante conhecido e descrito na literatura (2,17). Fatores biológicos, como flutuações hormonais durante o ciclo menstrual, gravidez, pós-parto e menopausa, estão associados ao risco elevado de depressão. Psicossocialmente, as mulheres são mais vulneráveis a estressores relacionados a papéis sociais, vitimização e status social desvantajoso, o que pode aumentar ainda mais a susceptibilidade à depressão (18). Pode-se dizer também que as mulheres são mais propensas do que os homens a reconhecer problemas psiquiátricos, a discutir essas questões com outras pessoas e a iniciar um tratamento voluntariamente (19).

Um hábito de vida investigado neste estudo foi a prática de atividade física. Observou-se maior prevalência de relato de diagnóstico médico de depressão naqueles que não praticavam atividade física. Outros autores reforçam que o estilo de vida sedentário pode levar à diminuição das atividades de vida diária, o que interfere na qualidade de vida, sendo a prática regular de exercícios um fator protetor para depressão em idosos (20,21).

Níveis mais baixos de escolaridade estão associados ao aumento dos sintomas depressivos. Isso se deve, em parte, ao fato de que pessoas com menor grau de instrução tendem a ter menos acesso aos serviços de saúde e a participar menos de atividades que estimulam as funções cognitivas e a interação social. Além disso, a educação tem sido reconhecida como um fator diretamente protetivo da saúde mental (22). 

Neste estudo, foi observado que idosos com até oito anos de escolaridade apresentaram maior prevalência em comparação aos não escolarizados. Tal achado, ainda que aparentemente contraditório à literatura, pode refletir descompasso entre expectativas e oportunidades vivenciadas por indivíduos com escolaridade intermediária, os quais, apesar de algum acesso à educação formal, podem não ter alcançado melhorias significativas em condições socioeconômicas. Essa condição pode acentuar sentimento de frustração e vulnerabilidade emocional. Esses resultados apontam para a importância de análises contextuais e mais aprofundadas ao se investigar a relação entre escolaridade e saúde mental na população idosa.

A discussão sobre depressão em idosos é complexa. O uso de autorrelato na avaliação de condições de saúde na população idosa pode ser benéfico, porém desafiador. O autorrelato permite que os idosos expressem diretamente suas percepções de saúde e bem-estar, o que é valioso, já que muitos sintomas podem ser subdiagnosticados por meio de avaliações clínicas tradicionais (23). De forma objetiva, a depressão nos idosos pode ser quantificada por meio de escalas validadas, a exemplo da Escala de Depressão Geriátrica, a qual determina critérios específicos para suspeita diagnóstica de depressão, ou até a necessidade de encaminhamento ao psiquiatra (24). 

É intrigante que este estudo tenha demonstrado altas taxas de sintomas depressivos em idosos sem relato de diagnóstico médico de depressão. Entre os 15,9% dos idosos que se sentiam deprimidos, apenas 37,3% relataram diagnóstico médico de depressão. Isso implica que 62,7% sentiam-se deprimidos, mas não relataram diagnóstico médico. 

Altas proporções também foram identificadas em outras perguntas como sentir que as coisas estavam mais difíceis do que costumavam ser antes, em que 77,9% de um total de 29,1% também não relataram diagnóstico médico. Esse comportamento se manteve em relação às demais perguntas relativas aos outros sentimentos investigados. Tais perguntas não necessariamente refletem depressão de forma direta, mas podem ser consideradas como possíveis indicativos para que os serviços de Atenção Primária possam intervir precocemente, de modo a prevenir a ocorrência de depressão. Categorizar tais sintomas em escores e aplicá-los no dia a dia pode ser um desafio (25). 

Para melhorar o manejo da depressão em idosos, é essencial que os profissionais de saúde combinem o autorrelato com as avaliações objetivas, por meio de escalas validadas e métodos de diagnóstico mais integrados, que podem incluir também exames de imagem e testes bioquímicos. Uma abordagem híbrida poderia levar à melhor identificação e tratamento de transtornos frequentemente negligenciados nessa faixa etária (23).

A dificuldade de relatar um diagnóstico médico de depressão pode estar relacionado ao fato de os idosos adotarem uma abordagem de autoajuda, hipoteticamente preferindo resolver seus problemas sozinhos, alinhando-se a narrativas culturais que valorizam a autossuficiência e a resiliência. Esse comportamento é reforçado pela visão de que os problemas de saúde mental são sinais de fraqueza pessoal, o que dificulta a aceitação de diagnósticos e tratamentos (26). A possível inacessibilidade dos idosos aos recursos de saúde pública também pode interferir e deve ser explicitada e abordada no planejamento de políticas públicas (15).

Ao esclarecer a importância do diagnóstico e de medidas de intervenção, é fundamental que os profissionais de saúde compreendam a necessidade de não manter o idoso subtratado. Este estudo revelou valores significativos de pacientes já diagnosticados com depressão que continuaram a apresentar sintomas depressivos. Essa situação pode ocorrer pela sobreposição dos sintomas depressivos com outras condições de saúde (27). 

A percepção da saúde, ou autoavaliação da saúde, é um dos indicadores a ser utilizado em pesquisas envolvendo idosos, e sua relevância se deve ao fato de que a percepção negativa da saúde pode ser um forte e consistente preditor de mortalidade (28). Em 2011, em Pelotas, 46,9% da população idosa teve a saúde avaliada como regular ou ruim, e 66,6% dos idosos com sintomas depressivos relataram percepções negativas sobre sua saúde (29).

É essencial que os profissionais de saúde e a rede de apoio estejam atentos às condições que aumentam o risco de desenvolvimento da depressão, bem como aos seus sinais e sintomas, rompendo com a percepção de que esses aspectos são naturais do processo de envelhecimento. Esses profissionais devem considerar o conjunto de fatores que impactam a vida e a saúde mental dos idosos e adotar abordagens mais abrangentes que reconheçam a multicausalidade das doenças (30), incluindo a depressão. Portanto, torna-se fundamental incorporar questões sociais e ambientais na análise, a fim de obter uma compreensão mais completa da realidade dessa população, além de direcionar esforços para a promoção de políticas públicas e o aprofundamento de estudos voltados ao grupo etário que mais cresce na pirâmide populacional.

Conclui-se que há diferença relevante entre o autorrelato de diagnóstico médico de depressão e a autopercepção de sintomas depressivos entre idosos brasileiros. A proporção de pessoas que referem sentir-se deprimidas supera aquela que relata ter recebido diagnóstico médico, o que evidencia possível lacuna no reconhecimento clínico da condição. Fatores como sexo feminino, sedentarismo e menor escolaridade mostraram-se associados à maior prevalência de sintomas depressivos. O autorrelato, apesar de valioso, pode ser limitado pela subnotificação ou pelo autoconceito dos idosos, o que reforça a necessidade de abordagem híbrida que combine autorrelato e métodos objetivos para o diagnóstico e manejo adequado da depressão.
